# Building and Sustaining Inpatient-Clinician Collaboration in Spinal Cord Injury Rehabilitation: A Case Example Using the Stoke Mandeville Spinal Needs Assessment Checklist (SMS-NAC) and Goal Planning Programme

**DOI:** 10.3390/jcm11133730

**Published:** 2022-06-28

**Authors:** Jane Duff, Lucy C. Grant, Helena Gilchrist, Kevin Jones

**Affiliations:** Department of Clinical Psychology, National Spinal Injuries Centre, Stoke Mandeville Hospital, Buckinghamshire Healthcare NHS Trust, Aylesbury HP21 8AL, UK; lucy.grant15@nhs.net (L.C.G.); helena.gilchrist@nhs.net (H.G.); kevin.jones8@nhs.net (K.J.)

**Keywords:** goal planning, goal setting, rehabilitation outcome, Stoke Mandeville Spinal Needs Assessment Checklist (SMS-NAC)

## Abstract

Goal planning is core for the delivery of the biopsychosocial model of rehabilitation and is commonly practiced in spinal cord injury (SCI) and other physical health settings. Despite a strong theoretical basis from several branches of psychology, evidence regarding specific practice, interventions and impact has yet to be established, with no universal standards in this area. Study One outlines the standards used at the National Spinal Injuries Centre (NSIC), Stoke Mandeville Hospital since the inception of the SMS-NAC and goal planning programme in 1989. The results outline the impact of a quality improvement project undertaken since 2016 and track the interventions used to improve inpatient care. Study Two reports on an international survey of rehabilitation measure usage and goal planning practice with inpatient adult and children and young people (CYP) with SCI. Respondents replied that inpatient presence at goal planning meetings only took place in 75% (adult) and 76% (CYP) of services, with more services indicating 4 or more members of the multidisciplinary team being present (85% and 90%, respectively). This paper demonstrates the gains that can be made when a structured quality improvement methodology is used and highlights the need for standards regarding goal planning in SCI rehabilitation to be developed.

## 1. Introduction

Goal planning and goal setting have been used in physical healthcare and spinal cord injury (SCI) rehabilitation for many years. Goal setting theory draws from behavioural psychology, which states that goals affect action [1]. Locke and Latham (1990), in their history of goal setting theory, consider there to be two influences: experimental psychology (intention and motivation) and management theory [2]. Duff (2007) summarises this literature in relation to spinal cord injury, commenting that “goal directedness is influenced by the value and significance of the goals and the individuals intention in achieving the goal” [3] (p. 223) and outlines the central role of social cognition and self-efficacy within goal choice, commitment and thereby performance [4]. Rauch and Scheel-Sailor (2015) comment that “goal setting is a process that includes the decision about what needs to be accomplished (the goal) and the formulation of a plan to achieve that desired result. It includes the identification of goal areas in different hierarchical levels and clarification of the corresponding expected outcome” [5] (p. 161). For the purposes of this article, the term goal planning is used synonymously with goal setting as an inclusive phrase to include any form of goal-directed rehabilitation activity.

Professor Paul Kennedy at the National Spinal Injuries Centre (NSIC) at Stoke Mandeville Hospital was an early implementer of systematised goal planning in SCI in 1989, finding through behavioural mapping that goal planning was an effective way to increase engaged rehabilitation activity and inpatient’s involvement in making decisions regarding their rehabilitation [6,7]. The centrality of individual (patient) needs and partnership with the multidisciplinary team (MDT) are fundamental values of the programme he developed and was influenced by the founding principles of Rehabilitation Psychology: namely, that emphasis should be placed on an individual’s assets; behaviour is a function of the person and environment; treatment should be comprehensive (physical and psychosocial); treatment should be tailored to the individual’s needs; the patient, to the degree possible, should have an active role in his or her own rehabilitation process [8,9] and led to the creation of the Needs Assessment Checklist (NAC) [10]. The NAC enabled inpatients to rate on admission to hospital their knowledge, skills and self-management needs, on a 4-point scale of independence, as part of a facilitated assessment by a member of the clinical team and thereby provide a framework for tailored goal planning. The assessment was repeated upon discharge as a measure of the rehabilitation outcome. People with Spinal Cord Injury (PwSCI) and clinicians helped co-create the NAC and a Plan, Do, Study, Act (PDSA cycle) was involved in the implementation of the programme [11], with goal setting starting on one ward in the NSIC and then progressing to involve the other 5 adult wards and children and young people’s (CYP) ward, with the creation of the Child Needs Assessment Checklist (ChNAC) [12]. Clinical practice guidelines for the adult goal planning programme were set following an initial series of audits between 1993 and 1995 [13,14] and concerned the timescale for the administration of the assessment NAC, commencement of goal planning (within two weeks of the NAC completion) and that the NAC be repeated prior to discharge to gauge outcome, with periodic audit of these [15]. The programme is founded on the values of 1. collaborative inpatient and clinician goal setting with the NAC domains being based on the key self-management domains for a PwSCI, rather than areas of professional practice (e.g., physiotherapy, medicine, nursing, etc.); 2. parity of esteem and skill gain for all levels of spinal cord injury, with both physical and verbal/instructional knowledge given equal weighting and reduce potential ceiling effects of other functional measures of outcome. In a systematic review of SCI outcome measures, Dawson, Shamley and Jamous (2008) commented “as the NAC and the SCIM each reflect somewhat different (although likely related) constructs, and are each applied in different ways, choosing between these instruments depends crucially on the purpose that any potential user has in mind” [16] (p. 778). The SCIM is used at the NSIC to benchmark and compare functional outcome on a national and international basis, and the NAC to understand the inpatient’s knowledge, skills and functional outcome as well as providing a direct relationship with goal and targets for rehabilitation [15], with the relative contribution of both in rehabilitation recently compared [17]. The 1999 version of the NAC underwent a major review in 2008, with clinicians who used the NAC in UK spinal centres contributing to the updated version. Individual items in the nine domains (activities of daily living, skin, bladder, bowel, mobility, wheelchair and equipment, community, psychological health and discharge co-ordination) were amended and aligned with the clinical practice of the time.

‘Spread’ and ‘sustainability’ are recognised as the bedrock for creating and maintaining improvements and supporting quality gain across healthcare [18]. The NAC has been shared widely throughout the UK and the world since its inception, largely through personal requests. This was agreed upon on a case-by-case basis, and a user log was not developed. An updated version was released in 2008 after a UK-wide review led by the first author and available to download through the Shirley Ryan Ability Lab (available online: https://www.sralab.org/rehabilitation-measures/needs-assessment-checklist; accessed on 14 May 2022) and the Skin domain of the NAC formed a basis for a French adaptation [19,20]. Further NSIC-based reviews of the NAC took place in 2015 and 2020. In 2015, a physical healthcare domain, an inpatient values assessment and the Appraisals of DisAbility: Primary and Secondary Scale short form (ADAPSS-sf) [21] were added (Appendix A outlines the breadth covered by the SMS-NAC from a case example). It was recognised that the lack of a central user log hampered the spread of this version and limited across-centre research; therefore, the NAC was renamed the Stoke Mandeville Spinal Needs Assessment Checklist (SMS-NAC) and copyrighted to ensure version control and promote comparative research, but remains unlicensed and publicly available through the correspondence address. A survey was conducted as part of the goal planning instructional course at the 57th International Spinal Cord Society Conference (ISCoS) [22] to gain an understanding of spread to aid cascade of future versions and regarding goal planning practice. The 2020 version of the SMS-NAC included major revisions of the skin, bladder, bowel care and psychological health domains (including replacement of the Hospital Anxiety and Depression Scale (HADS) [23], which had become licensed, with the Patient Health Questionnaire-9 (PHQ-9) [24] and Generalised Anxiety Disorder-7 (GAD-7) [25] to assess symptoms of depression and anxiety). This version has been shared and adopted by some UK spinal cord injury centres and with worldwide enquirers, although COVID-19 has impacted this cascade.

The Child Needs Assessment Checklist (ChNAC) was developed for use in paediatric spinal cord injury rehabilitation [12]. Similar to the adult-focussed SMS-NAC, the measure has been shared since its inception to enable version control and research, renamed and copyrighted SMS-NAC-Child (SMS-NAC-ch), though still unlicensed and publicly accessible through the correspondence address. It is a practical tool for planning and monitoring progress in CYP rehabilitation and for reflecting meaningful outcomes. It is a behavioural checklist consisting of 10 rehabilitation domains spanning a young person’s physical healthcare through discharge coordination. The SMS-NAC-ch is underpinned by treatment theory [26]. It is a developmentally sensitive measure that takes into account a young person’s physical and cognitive development and enables children and caregivers to reflect on the relative roles each takes within any particular rehabilitation activity. In this way, the measure allows the tailoring of rehabilitation both to a young person’s overall developmental level and also to their family, community and cultural context. In practice, the SMS-NAC-ch is completed with a child and caregiver by a clinician who knows the child and family well. The SMS-NAC-ch is completed at the beginning of the rehabilitation programme and is based on the respondent’s own views of their (or their child’s) skills and knowledge, and is then used to direct and target rehabilitation to the particular needs identified. The SMS-NAC-ch is then completed shortly before discharge from rehabilitation to provide an evaluation of progress and to highlight any outstanding issues. The SMS-NAC-ch is also useful clinically in tracking progress and growth throughout childhood and adolescence, and monitoring how a young person’s skills and independence develop as they age with their SCI.

The spread of the principles of goal planning and its application within spinal cord injury and neurological conditions has also been at the forefront of the development of the programme. Professor Kennedy and the first author, JD, provided workshops to UK SCICs between 1998 and 2003 and training for clinicians in neurological services on goal planning theory to map their own user group’s needs and create an assessment tool that captured user’s instructional knowledge and skills as well as functional ability.. Professor Kennedy also received a visiting fellowship from the New South Wales Government, Ministry of Science and Medical Research, Australia, in 2005.

Sustainability in healthcare has been found to be a significant challenge, with a systematic review of 125 studies acknowledging the paucity of research into healthcare improvements after initial implementation, and with many failing in terms of rigour, fidelity, detail regarding the adaptation of interventions over time, or consideration of cultural and organisational change [27]. An evidence review of the context for successful quality improvement found there to be macro (health system such as population demographic and location), meso (organisational such as culture and collaborative working) and micro (clinical team) factors that sustain change [28]. Duff (2008), in a chapter on goal planning in SCI, comments that “goal planning is a sophisticated psychological intervention which aims to impact at both the individual patient and systems level” [29]. Duff references the specific role appraisals of ‘manageability’, approach and avoidance from the Duff and Kennedy (2003) [30] model of SCI adjustment on the individual-level impact in relation to emotional reaction and long-term outcome. The systems (meso) level impact is aligned with goal setting theory and supports the biopsychosocial approach of rehabilitation. Multidisciplinary (MDT) rehabilitation recognises that “each professional team works independently of each other: working towards goals defined by their specific skills” (p. 12) with some collaboration on care, joint working and decision making [31]. It recognises that all members of the MDT bring skills to ensure goals are set at the right level of challenge and achievement, with hierarchical skill acquisition built in and “requires a collaborative view of rehabilitation from patients, multidisciplinary teams, commissioners and policy makers… the biopsychosocial rehabilitation model is now quoted as the ‘Gold Standard’ for informing clinical interventions, outcome evaluation, pathway design, and social interaction [31] (p. 11). The chapter includes a discussion of the other models: unidisciplinary, interdisciplinary and transdisciplinary. Such collaborative work is essential to enhance an individual’s appraisals and the development of self-efficacy following injury.

In terms of the micro level, Duff (2008) outlines a key element of goal planning to be “a shared process which is patient focussed and led” [29] (p. 71). The importance of the interconnected approach of goal setting by different clinical specialities, formulated around the clients’ needs rather than professional boundaries/delivery and centrality of psychological health, is emphasised in a range of case studies on goal setting using the International Classification and Functioning (ICF Case StudieS—15|Psychological Issues And SCI (Available online: icf-casestudies.org (accessed on 14 May 2022)), which also base intervention on the Folkman and Lazarus transactional model of stress and coping from which the Duff and Kennedy (2003) [30] model is derived. The case studies demonstrate the ICF assessment sheet, which captures both a ‘health professional’ and ‘patient’ perspective, as well as using the ICF Categorical Profile and Rehab-Cycle model, which emphasises shared decision making. Maribo et al. (2020), in a systematic review of qualitative studies of goal setting, found goal planning to support awareness of and adaptation to disability and promote autonomy, while also supporting adherence to treatment recommendations and rehabilitation satisfaction [32]. Recommendations following their review emphasise the essential nature of inpatient and family involvement from the beginning of goal planning, with the inpatient and the team having shared goals. In terms of the quality of the goals, Maribo et al. (2020) comment on the importance of relating goals to everyday life outside of hospital, with specific goals for discharge and hospital transition linking with a functional goal and in turn to the inpatient’s wider need or values, with equal emphasis placed on psychosocial components (family, employment, changed roles and independence) as there is on functional tasks such as transfers [32]. In a review of shared decision making (SDM) in first-time rehabilitation after SCI, SDM was found to be associated with better rehabilitation outcomes and especially recommended when preference-sensitive decisions are required [33]. Stigglebout et al. (2015) outline four steps of SDM: First, the healthcare professional (HCP) informs the patient that a decision is to be made and that at the patient’s opinion is important; second, the HCP explains the options and their thoughts about the advantages and disadvantages of the treatment being discussed; third, the HCP and patient discuss the patient’s preferences and the professional supports the patient to think through; fourth, the HCP and patient discuss the patient’s wish to make the decision, they make or defer the decision, and discuss follow up [34]. This process is highly relevant to goal setting in advance of the goal planning meeting. The first step takes place shortly after admission or when the Keyworker for goal planning meets with the inpatient to explain the programme and steps two to four being followed by individual clinicians ahead of a goal planning meeting so that joint goals can be brought. Furthermore, it has been suggested that goal setting inclusive of the person increases their physical gains [35], with measurable goals boosting skill acquisition [36].

Levack et al. (2006), in a review of goal planning with adults across a range of clinical conditions, found limited evidence for adherence but strong evidence that “prescribed, specific, challenging goals can improve patient performance in some specific clinical contexts” [37] (p. 739) and commented on the difficulty of the field in comparing goal planning programmes because of the variability in methodology. A Cochrane Review of goal pursuit in acquired disability was again impacted by the variation in terminology and methods and thus found only low quality evidence, concluding “the best evidence appears to favour positive outcomes (i.e., health-related quality of life, emotional status and self-efficacy) rather than physical ones” and said key research was needed to understand “how components of the goal setting process (such as how difficult goals are, how goals of therapy should be selected and prioritised, how goals are used in clinical practice, and how feedback on progress towards goals should be provided) contribute or do not contribute to better health outcomes” [38] (p. 2). A survey of goal planning was conducted alongside an instructional course at the 57th International Spinal Cord Society (ISCoS) [22], which indicated substantial variation in the fundamentals of the approach in SCI rehabilitation, such as whether goal planning took place as a team pursuit (i.e., involving four or more members of the rehabilitation team) and included inpatients and their families in the goal setting.

Collaborative goal planning is also recognised as a core component of rehabilitation practice with CYP. Many of the theoretical frameworks underpinning adult practice have relevance for CYP rehabilitation. A recent scoping review highlighted the particular relevance of social cognitive theory, self-determination theory, health action process approach theory, mastery motivation and goal setting theory [39]. However, the same authors found in a scoping review of 62 mobility-related rehabilitation studies that the theoretical underpinnings of goal planning interventions are rarely described in detail in the literature [40], highlighting the need for more and better quality research into an aspect of care that is widely commended and embedded within routine clinical practice.

The meso (organisation) level was considered as part of the sustainability of the initial development of the NSIC programme. Examples include a Goal Planning Strategy Group overseeing the programme’s development, clinicians from any professional background being able to be a Keyworker and provided with specific training for this role and PwSCI participating in the twice-yearly training since 2008. Further meso developments have taken place following the commencement of a regular audit of the programme in 2016 and are outlined as part of this paper.

Guideline or standard setting is often a key first step to enabling meso (system) level change, as it enables reductions in quality to be identified and improvements made. The goal planning programme at Stoke Mandeville, led by the first author, JD, since 2008, has undergone various stand-alone audits of the process, including patient experience (2003 and 2008) and staff experience (2003 and 2016) [14]. The fundamental values of the programme in relation to its founding values, goal achievement and correlational relationship of the NAC with goal planning and outcome were established and provided the first UK process map of rehabilitation and evidence of the individualised approach of the system by level of injury [15], with further audits in 2011 and 2015. This research enabled subsequent mapping to develop [41] and international audits [42] based on the findings. Outcome data from the NAC (now SMS-NAC) has also been used to evidence the impact of the programme, examining whether goal achievement varies for a range of potentially marginalised groups, including older adults [43] and people with pre-injury mental health needs [44], comparing outcome with respect to ethnicity [45], injury aetiology [46,47,48] and psychological need [49]. The breadth of the domains and items on the NAC (now SMS-NAC) has enabled self-management concerns such as skin knowledge skills development through rehabilitation to be examined [19,50,51] and issues such as pain [52] and return-to-work intentions to be tracked [53], with recent reporting of the mediating relationship that appraisals provide for mood and self-management on the skin and bladder domains [54]. For ease of comprehension, the NAC and ChNAC shall be referred to by their new names: SMS-NAC (adult) and SMS-NAC-ch (CYP) herein.

The NSIC programme has been accredited by the Commission for Accreditation of Rehabilitation Facilities (CARF), with the adult SMS-NAC and goal planning process in particular commended. In 2014, CARF reported, “The SMS-NAC is a truly exemplary assessment strategy and tool for evaluating both the knowledge and skills needed by persons with SCI to function successfully in the community. The SMS-NAC takes into consideration personal choice with respect to autonomy in managing one’s own care needs. The NSIC is especially commended for the extra efforts taken to validate the instrument and demonstrate its utility in a number of peer-reviewed research articles”. In 2018, the reviewers commented, “Incorporating the SMS-NAC, which facilitates patient engagement of the persons served and their caregivers in goal planning and direction, demonstrates a robust process and superior level of care. The goal planning programme is a detailed and comprehensive approach to goal creation and execution that is inclusive of the person served at every point of the process. The Keyworker is a unique position that utilises internal resources to advocate for the persons served and family throughout the length of stay”.

Hart et al. (2015) reviewed the role of ‘softer skills’ in change management: leadership, team building, culture, context and patient and family engagement in relation to sustained quality improvement [55]. Although the above audits and research have been useful in establishing the credentials and impact of the programme, in 2016, it was recognised that the programme needed regular review against its founding values to identify trends for assurance and equity in rehabilitation provision and to enhance its sustainability. Target setting for successful rehabilitation goal planning is as much the basis for this as it is for successful change management. This paper reports on two studies. Study one concerns the quality improvement project commenced by the NSIC in 2016, with information on the process of target setting, the consequent interventions used to improve the quality of the programme, and provides a case example of individual rehabilitation outcomes from an adult using the SMS-NAC. Study two reports on responses from a recent international survey of goal planning practice conducted by authors JD and HG.

## 2. Methods

### 2.1. Context

The National Spinal Injuries Centre (NSIC), Stoke Mandeville Hospital is a 102-bedded spinal cord injury centre, with 93 NHS England commissioned beds for adult service (providing 30% of the capacity across England, with a further 7 other commissioned centres providing the remainder) and is the leading provider for CYP, with 9 inpatient beds providing first-time rehabilitation and lifetime review as developmentally required. The adult service provides 24 acute care beds (6 for adults requiring ventilation and 1 CYP bed). The referral to catchment population is 1:35,000 and serves 5 major trauma centres with 31 associated district general hospitals. In fiscal year 2020–2021, new adult referrals were 128, new CYP referrals were 22 and re-admissions (a combination of adult and CYP was 204 (with fiscal year 2019–2020 comparisons of 110, 27 and 471—the latter was reduced in the most recent full fiscal year due to COVID-19). The service is part of Buckinghamshire Healthcare NHS Trust, which provides acute and community treatment for the county of Buckinghamshire, UK. The NHS England Spinal Cord Injury Clinical Reference Group oversees commissioning and service delivery.

Spinal cord injury services delivered by NHS England underwent a peer review between 2016 and 2017, with quality indicators for delivery being established (D13-16). This led to the NSIC reviewing its practice for the adult inpatient service and commencing the reported quality improvement project with a regular audit. There was a specific standard (D13-16-201 (adult) and 601 (child)) stating, “The SCIC should have a policy whereby a single named keyworker for the patient’s care at a given time is identified for each individual patient” and that “the SCIC should have a policy on a goal-orientated programme of care which includes that each patient should have a statement of rehabilitation aims and component elements to achieve those aims; that documented goal planning meetings should be held between the members of the MDT and the patient on at least the following stages: 1st should take place within 3 weeks of mobilisation; no less than 4-weekly thereafter; discharge planning takes place with staff, the patient and community personnel prior to halfway through the anticipated length of stay (D13-16-203 (adult) and 603 (child)).

### 2.2. Study One

The quality improvement project (QIP) for the adult inpatient programme commenced in June 2016. Prior to 2016, there were three programme standards. First, the SMS-NAC should be completed with an inpatient within two weeks of mobilisation (as the measure assesses user self-reported knowledge and skills; this timeframe enables some development of this). Second, goal planning commenced within 2 weeks of the completion of the SMS-NAC, and third, the SMS-NAC was repeated as a measure of outcome prior to discharge from hospital. The practice from the commencement of the programme in 1989 was to re-administer the second SMS-NAC about 4–6 weeks prior to discharge and thus provide a safety net to identify any outstanding goals and, where possible, complete a third SMS-NAC on the day of discharge. However, over time, the repeated administration of the SMS-NAC has merged into one; for the purpose of this study, the SMS-NAC completed prior to discharge was included.

Audits conducted in 2001 [15], 2011 and 2015, together with the department’s annual report, which captured data from 2008 regarding the repeated administration of the SMS-NAC, were used to set the initial targets for the QIP (Table 1). A known concern at the time was that from 2010, the pre-discharge ward was periodically closed to spinal cord injury inpatients and used for other conditions which impacted upon the completion of the second SMS-NAC, as this was part of the clerking in following transfer to this ward. Repeated administration of the SMS-NAC was not part of the routine practice of other rehabilitation wards and there was no mechanism to remind clinical staff to facilitate the completion of it.

The QIP audit takes place at four time points across the year and provides a ‘snapshot’ of practice: June, September, December and March, and is conducted by the Assistant Psychologist. In the first week of each quarter, a list of all current adult inpatients in receipt of first-time rehabilitation is sourced. Electronic inpatient notes are used to determine first-time spinal cord injury admissions. Inpatients whose discharge is delayed, whose rehabilitation has entered a maintenance phase or those who have yet to have commenced rehabilitation are excluded from that quarter’s audit. Data are gathered on nominated family information and Keyworker allocation and profession from the electronic patient file.

The second stage involves data regarding those who have commenced completing an SMS-NAC and goal planning since the last audit, with mobilisation to SMS-NAC and goal planning data recorded. Individuals who have not yet had a goal planning meeting or who have yet to complete an admission SMS-NAC will be listed as ‘Not started’, while inpatients who have not yet mobilised will be documented as ‘N/A’. For inpatients who have commenced goal planning, data will be collected on whether a goal planning meeting has been held within the last 4 weeks from the date of the current audit, as well as whether the date of their next scheduled goal planning meeting has been recorded in their inpatient notes. Following this, inpatient, family, Keyworker and MDT attendance at the most recent goal planning meeting (or second goal planning meeting where relevant regarding medical attendance) is recorded. Keyworkers can be any member of the MDT and it is an additional role to their primary clinical role, with specific training provided regarding the programme, advocacy and skills such as setting specific goals and targets. Most members of the MDT Keywork 1–2 inpatients at any point in time. The exception to this is the Clinical Psychology Team, who Keywork more and anyone with psychological complexity such as significant risk, mood disturbance or adjustment difficulties, as outlined in the NSIC Stoke Mandeville Psychological Care pathway [56] (p. 322).

Finally, the Assistant Psychologist will check each inpatient’s physical folder (which is at the bottom of the bed) and document whether the folder contains the most recent copy of their goal planning meeting notes (attendance is documented on this). Following final data checks, data is entered into an ongoing yearly tracker which is divided into each quarter of the fiscal year and outcome is reported and reviewed at the quarterly Goal Planning Strategy Group, with three of the main quality indicators (whether goal planning commenced within 4 weeks of commencement of rehabilitation; completion rate of the second SMS-NAC; and medical attendance at the second goal planning meeting) also being reported on the NSIC corporate dashboard.

### 2.3. Study Two

The goal planning survey was conducted online between 20 March and 21 April 2022 and was promoted through the first author’s membership of the social media platforms Twitter and LinkedIn and through direct and group email, the latter to the membership of the International Spinal Cord Society (ISCoS) through the society. ISCoS also promoted through its Twitter account and LinkedIn further to the first author’s post. The survey examined both adult and CYP goal planning practices and sought to ascertain current adoption and versions used of the adult and child SMS-NAC. Some of the content for the survey was based on the founding values of the NSIC goal planning programme, such as inpatient involvement in rehabilitation goal planning, presence of a quorate MDT (of at least 4 members), and a measure that captures verbal/instructional knowledge [15], and complemented the previous survey conducted by the first author (Duff, 2018, which was partially informed by the NHS England Peer Review quality indicators for goal setting). The current survey was extended to include goal planning practices with CYP, with the same questions used for both. Appendix B provides the survey questions.

The initial post on Twitter was made on the 20 March 2022, followed by the first author reposting on the 19 April 2022 to announce the survey close on the 21 April 2022 and again on the 21st to confirm that the survey was closing at midnight. Retrospective analytics found 771 followers of the first author on the 1st March 2022, with an increase of 18 followers by the 1 April 2022 and another 18 followers by the 1 May 2022; the number of followers on the days of the posts was unable to be obtained. The post on LinkedIn received 13 reactions and 1 comment from ISCoS, who subsequently reposted on their LinkedIn. Direct emails were sent to 61 email addresses (who were previous participants in the 2018 study or those who had requested information on the SMS-NAC) and a group email was posted to the members of ISCoS by the Society Administrator on 23 March 2022. The analytics for the posts on Twitter, LinkedIn and the Mailchimp group email are reported in Table 2, Table 3 and Table 4.

## 3. Results

### 3.1. Study One

The QIP identified that some key data were unknown when the project commenced. First, although an expectation that each patient had an identified Keyworker and was always present at the goal planning meeting, data was not routinely collected on adherence to these. Second, although numbers of the completion rate of the first SMS-NAC per year were reported, it was not known whether every patient admitted had completed an SMS-NAC. Targets of 90%, 100% and 95% were set for each of these, respectively.

The QIP also collected data about adherence to the original programme values to identify any improvements needed. These concerned: firstly, that goal planning is a collaborative process involving the inpatient, family member and clinicians and thus provide some evidence of shared decision making; and secondly, that goal planning meetings occur regularly (within every 4 weeks). Initial QIP targets for SMS-NAC completion were planned using the data from Table 1, with Table 5 reporting the outcome.

#### 3.1.1. Inpatient, Family/Friend and MDT Participation in Goal Planning

**Figure 1:**
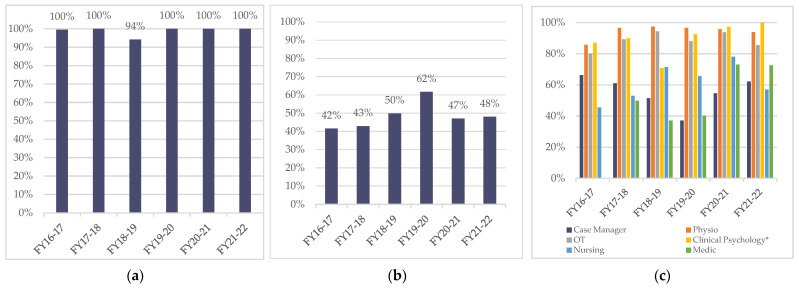
Goal planning participation: (**a**) Annual inpatient attendance at goal planning meetings; (**b**) Annual family attendance at goal planning meetings; (**c**) Annual MDT attendance at goal planning meetings. * *Clinical psychology attendance was calculated only for goal planning meetings where psychology was Keyworking*.

An underpinning value of goal planning at the NSIC is that it should be a collaboration involving inpatient, family and at least 4 members of the MDT and, through being based on the inpatient’s assessment of their skills and knowledge on the SMS-NAC, focussed on user’s needs. A primary feature is that goal planning meetings do not take place without the inpatient being present. QIP data revealed that inpatient attendance at goal planning meetings was 100% apart from the fiscal year 2018–2019, when it reduced to 94% (Figure 1a) but returned to 100% thereafter.

The attendance of a family or friend at goal planning meetings was also not recorded prior to the QIP. Accordingly, data were collected prior to the target of 50% being set in 2018. The target was achieved the following year (50%) and a higher achievement rate of 62% in the 2019–2020 fiscal-year audit (Figure 1b). Lower rates of family member attendance at goal planning meetings have been recorded since; however, this time period also overlapped with the COVID-19 pandemic and social restrictions for visiting.

The annual percentage of MDT attendance at goal planning meetings has collectively improved from 2016 until 2022 (Figure 1c). Physiotherapy attendance rose from 86% in fiscal year 2016–2017 to 94% in fiscal year 2021–2022, while Occupational Therapy from 80% attendance to 86% attendance. A target of 75% for nursing attendance was set on the basis of the audits in 2001, 2011 and 2015 because this was lower than other clinical groups. Nursing attendance rose from 46% to 78% by fiscal year 2020–2021 to achieve the target. However, as with family attendance, this also reduced during the COVID-19 pandemic and was 57% by the end of fiscal year 2022. Resource limitations of the NSIC clinical psychology team provide a clinical psychologist:inpatient ratio of 1:29 and a referral model is used for the service, which means all inpatients are unable to be assessed and a clinical psychologist largely only attends the goal planning meetings where they are Keyworkers. From 2016 to-date, an annual average of 22% of inpatients have been Keyworked by clinical psychologists, with only a small number of goal planning meetings being attended by the clinical psychologist when they are not a Keyworker. The NHS England Peer Review identified a gap in the medical team’s attendance at goal planning meetings. A target of attendance at the second goal planning meeting (when agreement is reached regarding the inpatient’s discharge date) was set and a target of 50% was introduced. The baseline measure was 37% and achievement increased year on year, with the target being reached by fiscal year 2020, which led to an increased target of 60% being agreed upon and subsequently achieved. Quality, Service Improvement and Redesign [57] tools were used to gain the achievements demonstrated and are outlined in the discussion. In relation to increasing nursing attendance, as demonstrated in Figure 1c, stakeholder consultation resulted in the implementation of a number of interventions, as outlined in Table 6. 

#### 3.1.2. Frequency of Goal Planning Meetings

**Figure 2:**
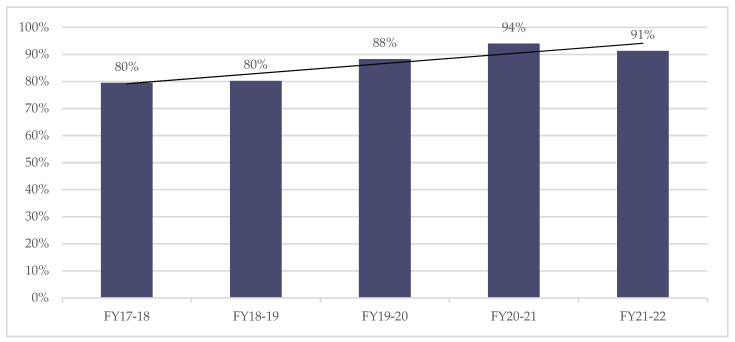
Annual percentage of inpatients who had a goal planning meeting in the last 4 weeks.

The original programme design aimed for a meeting every 2–3 weeks, so 3 weeks was the standard set at the commencement of the QIP. However, this was amended to within 4 weeks in 2018 to align with the guidance issued as part of the NHS England Peer Review. This allowed ‘slack’ in the NSIC system and facilitated greater capacity. This amendment was successful in delivering more frequent meetings the following year (88%) and subsequently enabled the maintenance of this improvement. Although the frequency reduced from 94% in 2020–2021 to 91% in the following fiscal year, there is nevertheless an upward trend, as shown in Figure 2. 

#### 3.1.3. SMS-NAC within 2 Weeks of Mobilisation

**Figure 3:**
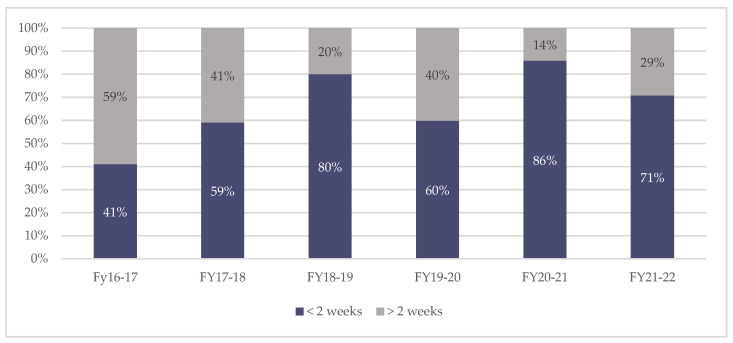
Annual percentage of SMS-NACs completed <2 weeks and >2 weeks from inpatient mobilisation.

One of the original standards was that the SMS-NAC be completed between the clinician and inpatient within 2 weeks of mobilisation. A target of 50% was set based on the 2001 audit and achieved in the fiscal year 2017–2018; this increased to 70% at the start of fiscal year 2020 and was also achieved. Figure 3 shows the variation in the percentage of SMS-NACs completed within this 2-week period. 

#### 3.1.4. SMS-NAC Administered Prior to Discharge

**Figure 4:**
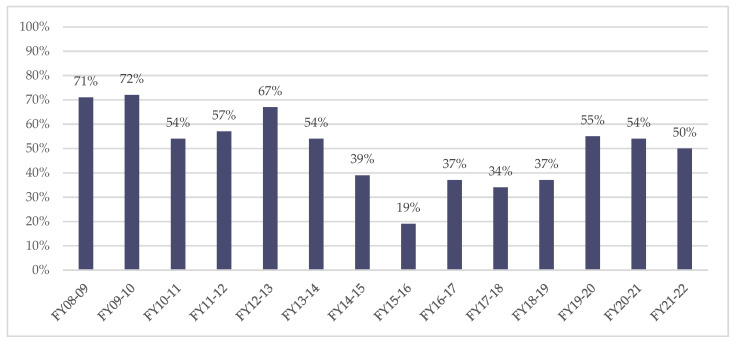
Annual percentage of second SMS-NACs completed of first SMS-NACs.

The administration of the SMS-NAC prior to discharge was significantly impacted by reduced bed capacity in the pre-discharge ward from 2010 (and ultimate removal of the commissioning of this provision), as the SMS-NAC was completed as part of the clerking in on this ward. As can be seen in Figure 4, at the start of the QIP, second SMS-NAC completion had reduced to 19% compared to 71% in 2008.

On commencement of the QIP, a target of 50% was set and an Excel spreadsheet was created with built-in coding, which enabled inpatients to be flagged as they approached discharge and reminders provided at the weekly Consultant/MDT review meeting. This led to an 18% increase the following year. In 2018, following nudge theory principles [58], individual email reminders were sent by the MDT/Consultant treating team to show progress. This led to some teams developing healthy competition to maintain their record of no outstanding second SMS-NACs, to the extent that there has been an overall 31% increase since the commencement of the QIP. The target was increased to 55% in 2020; however, the completion rate was reduced to 50% in the most recent fiscal year.

#### 3.1.5. Other Quality Standards and Target Increases

The regularity of the QIP audit has enabled the team to support other quality initiatives, such as when the Keyworking capacity of specific disciplines of the MDT has reduced due to staffing pressures to alleviate this gap. The communication cascade of goals through electronic and paper-based notes (available at the bedside to influence direct care provision) has been evaluated.

As indicated, when a target has been reliably established and embedded in clinical practice, an increased target has been set to further drive quality improvement and inpatient experience. Table 6 outlines the interventions and target settings since the QIP.

Other examples of quality improvements for inpatient experience have included producing leaflets with individual patients’ accounts of SMS-NAC and goal planning outcomes, aiming to connect newly injured adults with their potential outcome. An annual report of the programme has commenced, and a short film to explain the goal planning process and to model the collaborative nature of goal setting was produced to coincide with the launch of the SMS-NAC 2020. This is provided prior to admission to future inpatients as part of the outreach programme and is available on—Stoke Mandeville Spinal Needs Assessment Checklist and Goal Planning Programme (SMS-NAC)—YouTube—https://youtu.be/yPt7cvqzSKk, accessed on 14 May 2022) YouTube, with a longer version being used to support staff SMS-NAC and Keyworker training. Furthermore, a ‘My Health’ session with a clinical psychologist and three SCI user charities commenced in 2021 to discuss lived experience examples of self-management and advocacy. This was in part to connect inpatients with the recent development of social prescribing and personalised healthcare model in the UK (NHS England—Personalised Care Institute) [59].

#### 3.1.6. Case Study Using Adult SMS-NAC and User Testimony

The introduction outlined group research regarding the impact of the SMS-NAC and goal planning in inpatients’ rehabilitation outcomes from admission to discharge. The following provides a case example of the knowledge and instructional skills goals that can be set for someone with a cervical spinal cord injury and separate user testimony about the impact of gaining such skills, enhanced knowledge and value of collaborative involvement.

**Figure 5:**
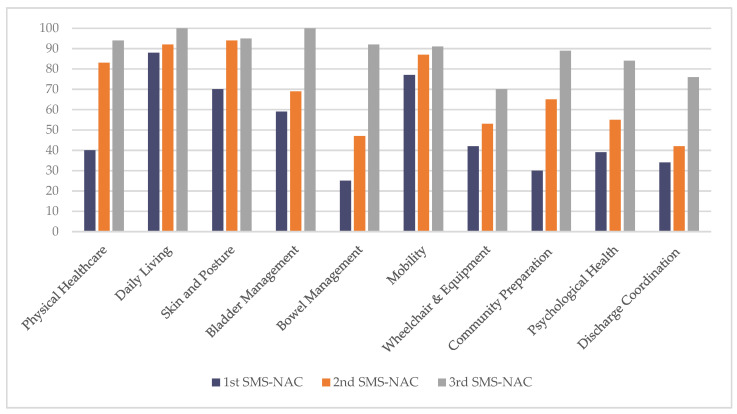
Petran’s first, second and third SMS-NAC percentage scores across outcome domains.

‘Petran’ was admitted to the NSIC following a fall at home, where he sustained a C4 complete spinal cord injury. He was transferred to the NSIC after treatment in a major trauma centre and his local general hospital. Petran had experienced poor care when waiting for admission to the NSIC and he was very clear from the outset of his admission that he wanted to gain verbal independence and instructional skills to be able to live well with care, although as can be seen from his initial SMS-NAC, he experienced difficulties with his mood and conceptualising this. His first SMS-NAC indicated a lack of knowledge or verbal instructional skills in bowel management, wheelchair mobility, community preparation and discharge coordination domains (Figure 5; Appendix A).

Because of difficulties in obtaining an accessible property for Petran in his home area, transfer to a care home was considered, and a second SMS-NAC was completed. The second SMS-NAC is usually completed about 4 weeks prior to discharge and is often a helpful review of the skills someone has gained while also providing a useful safety net for the PwSCI and team to look at the further goals needed. 

A third SMS-NAC was completed on discharge as a measure of his final outcome and significant improvements were evidenced across all 10 domains. In particular, the most substantial improvements were shown in the domains where Petran had initially indicated the least knowledge and independence at admission: bowel management (25% to 92%), community preparation (30% to 89%) and discharge coordination (34% to 76%), with his psychological health (as measured by the HADS, ADAPSS-sf and Perceived Manageability Scale (PMS) [60] and items asking about sexual health and carer needs) improving from 39% to 84%.

Qualitative user testimony about the programme was collected as part of the patient experience leaflets developed for users and explanatory and training films. The following are some excerpts:

“As my level of injury means I have very limited hand and arm movement, I believed I could not to be involved with very much. How wrong I was. As a more mature lady, I was worried I would not be offered the same rehabilitation programme. My age at the NSIC has never been a factor and I have been offered the full range of rehabilitation activities that a younger person would have had. My stay at the NSIC was not straightforward and physical setbacks severely knocked my confidence. But goal planning meetings, the support of my family and the team helped me to plan and set goals I wanted to achieve. I feel goal planning is a very empowering part of my rehabilitation. Through careful planning it showed me I could do things that I did not think were possible. NSIC staff encouraged me to take control of my own rehabilitation. Starting slowly, I was supported and able to set goals and achieve them. It took me a very long time to realise the importance of being verbally independent and being able to state how things should be done and how I would like them to be done. It was the best thing I could have achieved”

(S, 2017).

“Goal planning does help you understand about injury, and it gives you a more informed way of talking about your injury—in your head, things can seem like a mountain and it can help you bring it back into proportion. Initially it’s [SCI and rehabilitation] quite daunting, and you’re quite scared. You don’t really know what you need to do, and the staff help you to understand. Even things like understanding bladder problems and bowel problems, which you never even thought of being long term problems, the staff help you to understand that and manoeuvre your way through the system”

(M, 2019).

“The whole process allowed me to understand and focus on things that mattered, and to monitor them between meetings”

(A, 2019).

“I think my biggest personal achievement has been in my bladder and bowel care, where I have been able to make a great deal of progress, leaving the centre without a catheter and with full control. When that last piece of the puzzle fell into place, I was really proud of myself”

(E, 2019).

### 3.2. Study Two

**Figure 6:**
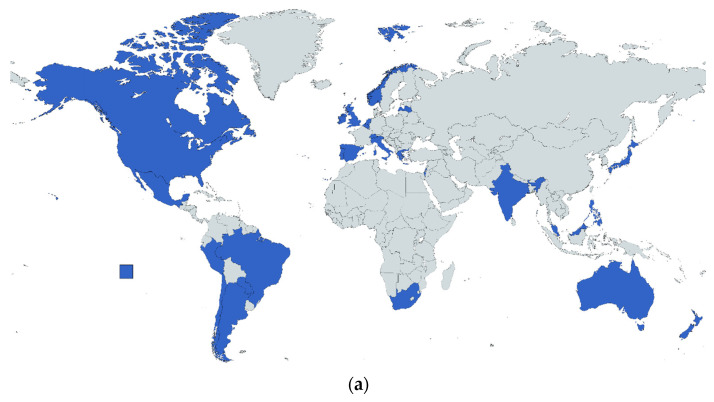

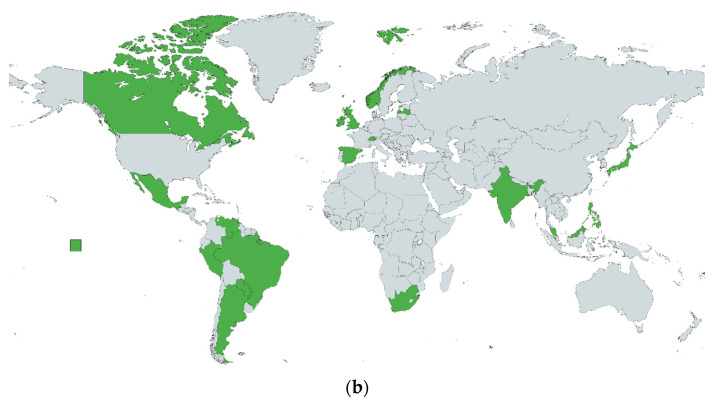
(**a**) Global reach of the 2022 Adult Goal Planning survey. (**b**) Global reach of the 2022 CYP Goal Planning survey.

Eighty-two participants from 54 SCI providers and 29 nations responded to the adult survey (see Figure 6a). There were multiple participants from 15 centres. Where the responses varied from clinicians working in the same provider location, the individual respondents were emailed to clarify their practice.

Twenty-two participants responded regarding CYP practices from 21 SCI providers and 18 nations (see Figure 6b). There were multiple participants from 1 centre who displayed inconsistencies in their responses. Responders from this site were emailed and provided with a clarification of their practice.

#### 3.2.1. Inpatient, Family and MDT Participation in Goal Planning

*Adult survey:* The results of the 2022 adult survey were compared with a similar survey conducted in 2018 [22]. The 2018 survey captured responses from 39 SCI providers across 15 nations. Little change was observed in family involvement or MDT attendance at goal planning meetings across sites (Table 7), with the majority of sites indicating this. A lower proportion of sites in 2022 ensured that inpatients were present at their goal planning meeting than in 2018 (Table 7).

Ten sites responded to the 2022 and 2018 surveys, which enabled changes across time to be identified. Two sites were removed from this analysis due to incomplete historic data. No change in goal planning practices was found in 50% of these sites. By 2022, 3 sites ensured that families were involved in the goal planning process, and 3 sites ensured that at least 4 members of an inpatient’s MDT attend goal planning meetings. One site that previously used the SMS-NAC had ceased using the measure and also reduced the frequency and application of their goal planning due to significant staffing and organisational changes.

*CYP survey:* In 2022, in comparison to adults, families were more often included in goal planning meetings. Likewise, a higher proportion of sites ensured the attendance of at least 4 members of the MDT at goal planning meetings for CYP than for adults. Little difference was seen in inpatient attendance at goal planning meetings between adult and CYP inpatients (Table 8).

The 2018 survey did not include questions relating to the rehabilitation of children with SCI. Therefore, no assessment of changes in practice over time could be examined.

#### 3.2.2. Frequency of Goal Planning Meetings

The UK quality indicators, spinal specification D13-16 standard, that goal planning meetings should take place no less than every 4 weeks were used. Adult survey respondents indicated that this tool is located in 75% of sites and 76% of sites working with CYP inpatients.

#### 3.2.3. Assessment Tool Used

Five respondents reported using the adult version and 4 reported using the child version of the SMS-NAC. Respondents for both were spread across 5 nations: India, Peru, the UK, Venezuela and Australia. 

Of the 48 centres that reported not using the SMS-NAC, 16 reported using an alternative tool and replied to further questions based on the assets of the SMS-NAC.

**Question 1: “We use a different holistic outcome tool that asks inpatients about their knowledge and skills as part of this tool.”**

In 2022, 33% of sites that work with adults and did not use the SMS-NAC reported using a different holistic outcome tool; this was reduced compared to 64% of respondents in 2018. This suggests that 59% of responding sites currently use no holistic outcome tool (either SMS-NAC or another), which incorporates inpatients’ self-reported knowledge and skills as part of their routine goal planning practice with adult inpatients. An even lower proportion of sites that work with CYPs reported using such a tool (29%).

Survey respondents reported using 16 different assessment tools to inform their practice (see Table 9). Five centres reported using 2 or more tools in combination. Of these 5 centres, 2 reported using a combination of tools that specifically target different domains of skills and knowledge to form a holistic picture of their inpatients’ skills and knowledge. Six sites reported using self-made tools but provided no further information. Two centres reported interviewing inpatients and/or family in answer to this question but did not report using any formalised measures.

A much greater variety of tools was reported in adults (16) than in CYP rehabilitation (3).

**Question 2: “This tool can assess verbal rehabilitation skills, enabling inpatients with high levels of SCI to have maximum outcome gain”**

Of those who worked with adult inpatients and reported using an alternative tool, 87% reported that the alternative tool used assessed verbal rehabilitation skills. This is an increase of 1% from the 2018 survey. Only 3 respondents who worked with CYP inpatients completed this question, all of which reported that their tool assessed inpatients’ verbal rehabilitation skills.

**Question 3: “The outcome/information from the assessment tool is used to set rehabilitation goals”**

Sixty-eight percent of respondents who worked with adults endorsed ‘Yes’, a reduction from 77% in the 2018 survey. Of the respondents who worked with CYP inpatients, 76% endorsed this item.

**Question 4: “We repeat the assessment tool we use at or near the end of rehabilitation”**

Seventy-two percent of respondents working with adults endorsed this item, which is slightly lower than the 2018 survey (77%) and comparable to respondents working with CYP (71%).

#### 3.2.4. Within-Site Discrepancy

Fifteen of the 54 sites were represented by multiple respondents. All but 2 of these sites displayed some level of within-site response discrepancy (Table 10). 

## 4. Discussion

Study One demonstrates the benefit of setting practice standards and targets and the impact of the systematic application of quality improvement methodology on the SMS-NAC and goal planning programme. The first author, JD, was trained in QSIR methodology [57] in 2018 and applied interventions such as stakeholder analysis, clinical engagement and supporting people through change as part of the QIP. The QIP also employed a range of meso- and miso-level interventions, as shown in Table 6. A meso-level intervention shortly after the commencement of the QIP was the interpretation of Buckinghamshire Healthcare NHS Trust (BHT) organisational values to align with the values of the programme, with the aim of supporting NSIC staff to connect with them in their daily work (Table 11).

The work of the Goal Planning Strategy Group (which includes representation from across the clinical workforce and a PwSCI) changed and became more action-orientated, with members agreeing meso- and micro-level interventions, consulting and promoting these with colleagues, with an example being the rationale for target increases. As can be seen from Table 6, a range of flexible interventions have been used to engage staff, including the regular sharing of outcomes and positive reinforcement for SMS-NAC completion and goal planning attendance. Without such engagement, improvement would not have been achieved or maintained.

Ten areas for improvement were selected, and the targets were set. Initial targets within eight of these were achieved with a higher target then being set, the exception being patient inclusion (whose target was 100% and could not be reset, although notably dropped below this on one occasion) and nursing staff attendance at goal planning meetings. The target for the latter was achieved twice but was not consistently across two cycles of the programme. It is important to reflect on the latter two years of the QIP in relation to the COVID-19 pandemic (a macro-level impact). The observed increase in nursing staff attendance in the first year of the pandemic was possibly contributed to by the reduction in elective admissions, thus creating greater availability of staff due to a total reduction in inpatient numbers. The subsequent fall to 57% is also likely to have been impacted by the pandemic, although in the opposite direction, and perhaps contributed to staff exhaustion [61] and high COVID-19 sickness rates as the pandemic progressed. The reduction in the SMS-NAC completion rate over the past year is also likely to have been impacted by the same staffing constraints, as well as lag in embedding change for inpatients with a short admission. Analysis of the data that took place in fiscal year 2019–2020 revealed that often inpatients whose admission was for a short duration (e.g., 3–4 weeks) were the gap in the SMS-NAC completion rate. A pathway review took place and a standard operating procedure was agreed upon for all adult and CYP admissions. Research has found that inpatients can fail to appreciate the therapeutic self-management training provided by nursing staff and consider this the prerogative of physiotherapists and occupational therapists [62]. Hill (2022) [63] identifies that the role of the rehabilitation nurse is better developed in the USA, Canada and Australia than it is in the UK and identifies “contributing to goal planning” (p. 43) as one of the core responsibilities. Van Diemen et al. (2021) suggested that making self-care training explicit (such as setting a goal about learning to wash or independence in bladder care) might enhance insight into the therapeutic role provided by nurses in rehabilitation [62]. From the SMS-NAC summary example given in Appendix A, such emphasis is part of goal planning at the NSIC. However, from the current study, the reduction of the nursing team in the goal planning meetings and the lack of visibility of this part of their role could mean that NSIC inpatients impacted by the absence also hold such views. Anecdotal experience of the first author, JD, is that inpatients place high value on all members of the MDT to be at goal planning meetings and consider when this happens that the relevance of the goals set are enhanced. It is positive that MDT attendance has increased overall since the QIP began and to note the substantial improvements made in medical staff attending the second goal planning meeting, both of which will have enhanced the potential for shared decision-making in the goals and targets set.

The pandemic has also had an impact upon family/significant other attendance at goal planning meetings. This is perhaps not an unusual finding for the first year of the pandemic, given the sudden introduction of restrictions on hospital visits for most of the 2020 fiscal year and only one visitor per patient per week, with allocated and timed slots for a significant length of time when visiting did recommence. It is positive that almost half of the goal planning meetings were able to be attended by nominated significant others across the pandemic. This outcome may represent the advantage provided by technology regarding increased flexibility (such as not needing to travel, taking time off work/arrange childcare, etc.) and may be the preferred choice for some inpatients and family/significant others going forward. However, further analysis is needed to understand the lack of improvement over time, given the integration of technology into many people’s lives during the pandemic. Possible contributors to the result may be the number of inpatients over 65 years of age (and the possible consequent age of their partner or friend, if nominated), which has been a noted trajectory for some years [64]. The QIP did not record the details of the nominated family/significant other, however it may be that concerns associated with reduced familiarity, access or desire to use technology impacted this. A further confounding aspect is that the percentage is derived from the total number of inpatients and that this does not take into account those who do not wish their family to be involved or who do not nominate or have family or a significant other. The QIP going forward will endeavour to identify these aspects to more fully understand this issue and increase inclusion when desired. 

In addition to family/significant other attendance, the repeated administration of the SMS-NAC has not shown resilience over the course of the pandemic (reducing from 54% to 50%); this is a significant concern and needs renewed focus going forward. Four of the selected areas of improvement demonstrated resilience during the pandemic, with achievement remaining above the most recent target set: SMS-NAC being completed within 2 weeks of rehabilitation starting*; goal planning commencing within 2 weeks of the SMS-NAC completion*; goal planning commencing within 4 weeks of rehabilitation*; and medical attendance at goal planning meetings. Although it may seem that those indicated by * are commenting on the same data, fine analysis during the QIP revealed that initial quarterly progress was either made in early completion of the SMS-NAC and delayed commencement of goal planning or late completion of the SMS-NAC and early commencement of goal planning. To track this at an organisational (meso) level and for ease of reporting to the NSIC board, the overall target of goal planning commencing with 4 weeks of rehabilitation was added. 

The survey data in study two reveals some consistency of goal planning practice with regard to MDT involvement. The question regarding family involvement requires further investigation, as it included both presence and being sent information and as such does not directly compare with the QIP data, which just recorded presence. It is also important to note the methodological limitations in the introduction, such as a common language regarding what is meant by goal planning, may impact the conclusions from this survey, as may participant self-selection bias. Most notable from the responses are the lack of universal inclusion of inpatients in the adult and CYP goal planning meetings, and the reduction of inclusion between the adult surveys of 2018 and 2022 suggests a worrying trend. The reason for the reduction is unknown, but it is hoped that this paper will stimulate concern and a reversal of this trend and perhaps provide the opportunity for standard setting and a common approach for goal planning practice in SCI rehabilitation to develop. The within-site variation in response, though largely resolved through further investigation, perhaps provides further weight to the need for standards or guidance in this area or highlights the difference between intention and experience, which is further commented on later. 

A variety of measures were identified as being holistic; however, on reflection, this is potentially a term that requires more precise definition. Significantly fewer measures were identified for CYP than for the adult population. This may in part be reflected by the lower frequency of SCI sustained in CYP and the consequent paucity of measures for this population. This is underlined by a recent publication that had one chapter on paediatric SCI measures and one on caregiver measures compared to nine chapters on measures for adults with SCI, many of which were either not relevant or unvalidated for a CYP population [65]. There are important gaps in understanding the needs and outcome for CYP with SCI and this is an area that requires attention [66], which the developmentally-targeted SMS-NAC-ch can support. The survey question regarding verbal instruction was phrased so that the reply was in the context of the alternative measure identified in the previous question, which limited the response pool. Similarly, this question would also have benefited from a more precise definition or an example being given of verbal instruction, such as “Can you (or do you instruct others to) dress your upper body?”, as on the SMS-NAC. As can be seen from the user testimony of ‘S’, inpatients place high value on the SMS-NAC summary, identifying instructional skills that they can develop during rehabilitation and that are operationalised through goal planning. In particular, a review of the summary graph at the goal planning meeting (Figure 5), which takes place after the second SMS-NAC, makes explicit the progress made as the SMS-NAC’s weighting gives parity for all levels of SCI. Eaton et al. (2022) found this to be a particular asset with an effect of improvement over time for the SMS-NAC of physical health, skin and posture management, bladder management and bowel management domains for inpatients with cervical injuries, with the smallest SCIM showing the smallest improvement for these inpatients [17]. These domains have the greatest impact with regard to PwSCI’s long-term health, with skills and knowledge in these areas preventing the development of secondary health conditions and rehospitalisation. Therefore, being able to understand outcomes at the end of rehabilitation regarding these skills is critically important.

Question 4, regarding repeated administration, indicates that many services aim to evaluate outcomes; however, it is not known whether this is an aspiration that is supported by adherence data. As can be seen from the second SMS-NAC completion rate between 2008 and 2016, service changes can mean something remains a value but can be reduced in real-time clinical practice. All the survey questions asked about intention rather than audited practice, which from the data in Study One can be seen to yield difference. The NSIC is fortunate, given its bed capacity for admissions, to have the numbers to examine statistically significant outcomes. However, between 2015–2020, only 215 of the 657 first SMS-NACs were repeated at discharge [49,54]. As can be seen from the QIP, there have been substantial improvements in the administration of this; however, the reduction remains a concern and an area requiring ongoing improvement, both in terms of the group outcome data that this yields and the individual impact for the inpatient. For the PwSCI, there is a dual purpose of being able to see the progress they have made and the second administration providing the essential safety net for identifying remaining skills and knowledge prior to discharge. One of the aims of ascertaining usage of the SMS-NAC and the version used through the survey is to enable across-centre research and build on previous publications examining demography and outcome in vulnerable groups.

One of the features of SMS-NAC is its correlational relationship with goal planning [15]. The responses to question 3 indicate that the assessment goal planning outcome relationship is a gap for many of the current measures used by the participants. Goal planning theory suggests that elements such as specificity, level of difficulty and ownership can impact performance and consequently self-efficacy. Duff et al. (2004) in the NSIC audit found that 72% of goals were achieved after the first goal planning meeting, with a subsequent achievement rate of 68% and overachievement rate of 2%, suggesting that the goals set at that time were of sufficient challenge [15]. Due to brevity, the survey did not ask participants about the technicalities of the goal planning undertaken. Goal achievement is one measure, but relatively crude, and goal planning practice at the NSIC and elsewhere would benefit from formally assessing a range of concerns regarding user experience. These could include someone’s rating of participation or involvement in shared decision making, confidence in ability to achieve the goal set and whether the goal maps on to their values. Munce et al. (2016) found there to be a discrepancy between the meaning of self-management between individuals with SCI (and their caregiver dyads) compared to clinicians and rehabilitation managers, and identified a research gap in what constitutes internal and external attributions for self-management [67]. Likewise, the received teaching experience of users in relation to self-management skills acquisition in rehabilitation requires greater understanding and interrogation. Van Diemen et al. (2021), similar to Munce, interviewed inpatients and found differences between user and clinician weighting regarding the self-management themes identified [62]. 

There are several future research directions for the SMS-NAC and goal planning programme at the NSIC, as well as broader developments needed in SCI. The SMS-NAC is a static picture on admission and discharge of knowledge, skills and functional ability. Research grant applications have been submitted (though to date have been unsuccessful) to make it into a live tracker and phone-based app so that inpatients can record gain, such as their increased knowledge on a given item when self-management training has been received or a functional goal achieved. The Patient Activation Measure (PAM) is the recommended measure of the Personalised Care Institute (PCI) in the UK [68] with emerging evidence of its ability to discriminate and plan intervention to improve self-rated health [69], associations for activation with improved mood, reduced healthcare cost as activation increased and increased healthcare cost when activation reduced [70]. Pairing of the SMS-NAC outcome with this assessment could be used across someone’s lifespan to support increased confidence after discharge (the PCI model recommends structured health coaching or motivational interviewing to increase confidence and activation) and could also help identify reductions of knowledge/confidence or changed clinical practice as people age with injury. There is evidence of significant vulnerability to secondary health conditions after discharge from rehabilitation, with 31% of PwSCI readmitted to hospital within the first year of discharge [71], and with consistent difficulties of urinary tract infection and skin concerns being reported as well as some vulnerability factors emerging, such as having a higher and more complete injury [72], being of younger age, a woman, unemployed or retired [71]. An intervention that builds on self-care knowledge, such as health coaching, particularly those that involve peers as coaches, has been found to be particularly beneficial [73,74] and could have a significant impact on supporting transition from hospital and long-term health. Currently, a short form of the SMS-NAC is used with PwSCI who are readmitted to ascertain knowledge and skills change, with the full measure and goal planning commencing if a score of 3 or more of the 12 items indicate a reduction, and incorporating the PAM and peers alongside goal planning would be advantageous. As well as the quality of inpatient experience of goal planning needing consideration, the psychological health benefits and understanding of psychological factors that influence improved self-management outcomes need to be identified. Research has commenced investigating interactions between mood, appraisals and self-care gain on the skin and bladder domains of the SMS-NAC [54] and the team has implemented generic psychology self-care goals (so that individual therapy confidentiality can be maintained, for example, in Appendix C). Both will help enhance the biopsychosocial goals set.

This paper demonstrates the gains that can be made when structured quality improvement methods are applied to SCI rehabilitation and the value of setting standards for goal planning practices. All aspects of the NHS Institute for Innovation and Improvement Sustainability Model [75] have been delivered as part of the QIP. However, quality improvement on this level and for the future requires continued effort and as has been seen, macro-, meso- and miso-level changes can easily lead to reductions in inpatient care. There will be a formal review of the programme in relation to the sustainability model by the Goal Planning Strategy Group to identify potential threats and areas for further development. The survey revealed a lack of universal inclusion of inpatients. This is a serious concern, given that they are a key contributor to goal achievement and, thereby, goal planning. This is especially so given that the cornerstone of rehabilitation is developing self-care skills to live life well, with the consequent positive impact on quality of life, self-efficacy and reduction in secondary healthcare complications/rehospitalisation. This report highlights a range of possible improvements in SCI goal planning in rehabilitation, but inpatient:clinician collaboration in goal planning is perhaps the most fundamental place to start.

## Figures and Tables

**Table 1:** 2008 annual report data showing repeated administration of the SMS-NAC and the percentage proportion of second SMS-NACs completed by first SMS-NACs.

UK Fiscal Year (5th April–4th April)	Number of 1st SMS-NACs Completed	Number of 2nd SMS-NACs Completed	Total SMS-NACs completed	2nd SMS-NAC % of 1st SMS-NAC
08–09	136	96	232	71%
09–10	145	104	249	72%
10–11	142	77	219	54%
11–12	151	87	239	57%
12–13	119	80	199	67%
13–14	134	72	206	54%
14–15	126	49	175	39%
15–16	126	24	150	19%

**Table 2:** Twitter analytics from survey Twitter posts ^1^.

Twitter Analytics	
Impressions	2921
Followers	789
Total engagements	110
Detail expands	31
Link clicks	25
Likes	21
Retweets	16
Profile clicks	13
Replies	4

^1^ Definitions: Impressions—number of times the Tweet was seen on Twitter; Followers—number of followers author JD had at the time of Tweeting survey (up to February 2022); Total engagements—times people interacted with the Tweet on Twitter; Detail expands—times people viewed the details of the Tweet; Link clicks—clicks on the URL which led to the survey; Likes—number of times people liked the Tweet; Retweets—number of times the post was retweeted; Profile clicks—number of clicks to the first author’s name/profile; Replies—number of replies to the Tweet.

**Table 3:** LinkedIn analytics from survey posts.

LinkedIn Analytics					
Top Industries		Top Professions		Top Locations	
Buckinghamshire NHS Trust	34	Mental Health Professionals	39	London Area	36
NHS England	5	University Professor	14	Greater Oxford Area	14
Wellspect HealthCare	4	Executive Director	13	Arnhem-Nijmegen Region	9
Spinal Injuries Association	4	Medical Assistant	11	Greater Chicago Area	7
Irwin Mitchell	4	Business Strategist	11	Greater Adelaide Area	6
Buckinghamshire Council	3	Physician	11	Manchester Area	5
Stewarts	3	Nurse	10	Greater Gothenburg Area	4
Sint Maartenskliniek	3	Salesperson	10	Greater Stockholm Area	4
Oxford Health NHS Foundation Trust	3	Human Resources Specialist	8	Greater Portsmouth Area	4
	**63**		**127**		**89**

**Table 4:** Mailchimp ISCoS analytics from survey posts.

Mailchimp ISCoS Analytics	
Recipients	1026
Total opens	463
Unique opens	77
Total clicks	198
Unique clicks	77

**Table 5:** Annual report data from FY16–17 to FY21–22 show the number of first SMS-NACs completed, the number of second SMS-NACs completed, and the percentage proportion of second SMS-NACs completed by first SMS-NACs.

UK Fiscal Year (5th April–4th April)	Number of 1st SMS-NACs Completed	Number of 2nd SMS-NACs Completed	Total SMS-NACs Completed	2nd SMS-NAC % of 1st SMS-NAC
16–17	155	58	213	37%
17–18	116	40	156	34%
18–19	155	57	212	37%
19–20	176	97	273	55%
20–21	137	74	211	54%
21–22	151	76	227	50%

**Table 6:** Targets for the quality improvement project and interventions from 2016.

Standard	Audits 2001; 2011; 2015	TARGET	FY16–17 **	TARGET	FY17–18	TARGET	FY18–19	FY19–20	TARGET	FY20–21	TARGET	FY21–22
Each inpatient to have identified Keyworker	*	90%	93%	95%	93% ^1,4,5^		91% ^7^	90% ^14^		95% ^19,21,22,24,25^		90% ^28^
SMS-NAC completed	*	95%	92%		81% ^1^		89% ^7^	96%	100%	80% ^19,23,25^		72%
SMS-NAC within 2 weeks of mobilisation/start of rehabilitation †	(52%, 47%, 26%)	50%	41%		59% ^1^		80% ^7^	60%	70%	86% ^19,23^		71%
Goal planning within 2 weeks of SMS-NAC †	(62%, 70%, 63%)	70%	46%		69% ^1,3^		77% ^7^	63% ^18^		80% ^24^	75% ^14,19^	76%
Goal planning within 4 weeks of start of rehabilitation †	*	70%	45%		69% ^1,3^		62% ^7,10^	75% ^18^		79%	75%	77%
SMS-NAC repeated prior to discharge	(78%, *, 19%)	50%	37%		34% ^1,3^		37% ^7,9,12^	55%		54%	55%	50%
Patient inclusion	*	100%	100%		100% ^2,6^		94%	100% ^15,24^		100%		100% ^27,29^
Family/Significant other inclusion	*		42%		43%	50%	50% ^7^	62% ^15,17^	60%	47% ^26^		48%
Medical attendance	*		*		*	50% ^11^	37%	40% ^16^		73%	60%	77%
Nursing attendance	(68%, 53%, 50%)	75%	46%		53% ^1,8^		71% ^7,12,14^	66% ^13, 15, 16^		78% ^20,22,23^		57%

* data/outcome for this was not known / collected prior to the commencement of the 2016 quality improvement project. ** FY16-17 incomplete dataset due to the implementation and embedding of the standards process. † ‘snap shot’ data from quarterly audits. All other outcomes report on full fiscal year datasets. ^1^ 2017: Goal Planning Annual Report Commenced (meso level). ^2^ 2017: BHT CARE values interpreted to align with the programme (meso level). ^3^ 2017: Key quality indicators of goal planning commenced within 4 weeks of commencement for rehabilitation and second SMS-NAC added to NSIC score card. ^4^ 2017: Audit data cascaded via NSIC audit meeting and quarterly newsletter to staff (miso level). ^5^ 2017: SMS-NAC and goal planning clinical competency framework developed—staff awarded levels of competency certificates (miso level). ^6^ 2017: Patient experience and outcome leaflets commenced (miso level). ^7^ 2018: First author, JD, did Quality, Service Improvement and Redesign (QSIR) training with ACT Academy, NHS Improvement (meso level). ^8^ 2018: Bespoke ward goal planning meeting timetable (miso level). ^9^ 2018:SMS-NAC outcome reminders sent (meso and miso level). ^10^ 2018: Goal planning meetings amended to take place every 4 weeks (previously 3). ^11^ 2018: Revised timing for second (medical staff) attending GPMs (miso level). ^12^ 2019: Lead Nurse observes goal planning meeting (meso and miso level). ^13^ 2019: Nursing team survey of the programme (miso level). ^14^ 2019: One nurse to be a Keyworker for one patient on each ward (meso and miso level). ^15^ 2019: Brief patient introduction and one-hour staff skills training film created (meso and miso level). ^16^ 2019: Goals set at the GPM are discussed at the medical and nursing handover (miso level). ^17^ 2020: (January) Front page of the SMS-NAC amended to record inpatients nominated significant other for goal planning meetings (meso level). ^18^ 2020: (January) Goal planning process amended with launch of the SMS-NAC, January 2020, with the new summary structure putting discharge information before other domains (meso level). ^19^ 2020: Standard operating procedure consultation commenced to streamline the process and meet short-term admission SMS-NAC and goal planning gaps (meso and miso level). ^20^ 2020: Ward manager to Keywork and be supernumerary back-up support for nursing KW for the rota (meso and miso level). ^21^ 2020: Band 4 nurses can Keywork, training provided (miso level). ^22^ 2020: Involvement of Practice Development Nurses in SMS-NAC and Keyworker training (meso level). ^23^ 2020:SMS-NAC foundation skills training (miso level). ^24^ 2020: NSIC Patient Flow meetings commenced (meso level). ^25^ 2020: Quarterly excellence award for staff (cc ward manager) for the number of SMS-NACs completed (meso level). ^26^ 2020: Strategies as a consequence of COVID-19 to return family attendance to above 60%. ^27^ 2021: Monthly ‘my health’ patient information session and making the move leaflet to aid discharge transition (miso level). ^28^ 2021: Keyworker training (for those who are eligible) to be a mandatory part of the appraisal (meso level). ^29^ 2021: Generic psychology self-management goals documented and included as a target for the NSIC Clinical Psychology Team Quality Improvement weekly huddle (meso level).

**Table 7:** Inpatient, family and MDT participation in goal planning across sites in 2018 and 2022 for adult inpatients.

	Proportion of Sites	
	2018	2022
Family involved either through attending goal planning meeting or being sent information on goals set	87%	85%
At least 4 members of MDT attend goal planning meetings	85%	85%
Inpatient present at goal planning meetings	90%	75%

**Table 8:** Inpatient, family and MDT participation in goal planning across sites for CYP and adult inpatients in survey.

	Proportion of Sites	
	CYP	Adult
Family involved either through attending goal planning meeting or being sent information on goals set	95%	85%
At least 4 members of MDT attend goal planning meetings	90%	85%
Inpatient present at goal planning meetings	76%	75%

**Table 9:** Number of adult and CYP centres and the assessment tools used.

	No. of Centres	
Tool	Adult	CYP
Spinal Cord Independence Measure (SCIM)	9	3
Functional Independence Measure (FIM)	5	1
American Spinal Injury Association Impairment Scale (AIS)	3	
International Classification of Functioning, Disability and Health (ICF) Rehabilitation set	3	
Appraisals of DisAbility Primary and Secondary Scale (ADAPSS)	1	
Canadian Occupational Performance Measure (COPM)	1	
Goal Attainment Scale (GAS)	1	
Nottwiler Outcome Measurement	1	
Oswestry Disability Index (ODI)	1	
Rehabilitation Activities Profile (RAP)	1	
Reintegration to Normal Living Index (RNL)	1	
Spinal Cord Injury Secondary Conditions Scale (SCI-SCS)	1	
Utrecht Scale for Evaluation of Rehabilitation Participation (USER)	1	
Valutazione Funzionale Mielolesi (VFM)	1	
WHO Quality of Life-BREF (WHOQOL-BREF)	1	
Self-made tool	6	1

**Table 10:** Within-site response discrepancies.

Question	Frequency of Within-Site Discrepancies
We use a different holistic outcome tool that asks inpatients about their knowledge and skills as part of this tool	7
This other outcome tool can assess verbal rehabilitation skills, enabling inpatients with high levels of SCI to have maximum outcome gain	5
We ensure the information from the outcome/assessment tool is used to set rehabilitation goals	4
We repeat the assessment tool we use at or near the end of rehabilitation	4
We ensure all, or at least 4, members of the MDT attend the goal planning meeting	4
We ensure multidisciplinary goal planning meetings take place regularly during rehabilitation as needed, but as a minimum an average of every 4 weeks	3
We ensure the inpatient is always present at the multidisciplinary goal planning meeting	2

**Table 11:** BHT’s organisational values.

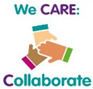	*Comprehensive multidisciplinary team work collaboratively planning rehabilitation goals with the patient and their family*
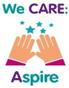	*A focus on the patient’s strengths, setting aspiring personalised goals facilitating patient and family adjustment to SCI*
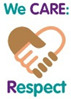	*Patient’s values and experience are respected through setting personally meaningful goals*
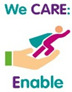	*Skills teaching and education enabling lifelong self-management skills, with patients becoming experts by experience through their rehabilitation and promoting future health*

## Data Availability

The data used in the current study were part of a standard clinical database that contains identifiable patient information and were therefore not publicly available. Pseudonymised data can be made available upon reasonable request to Buckinghamshire Healthcare NHS Trust.

## Appendix A. Petran’s First SMS-NAC Summary

Patient Goals and Information

- Petran enjoys being active.
- Petran would like to spend some time out of the hospital with his sister.

Discharge Co-ordination

- Petran needs to be made aware of the services provided by Spinal Out-Patients.
- A provisional discharge date should be set at the second goal planning meeting.
- Petran’s NSIC case manager should assess/discuss his discharge needs, including legal advice, if applicable.
- Petran needs to be given support to apply for all the appropriate benefits.
- Petran was living in privately rented accommodation at the time of his injury. Petran requires rehousing; a housing needs report and application should be made to his housing department.
- Petran should be given the opportunity to use the NSIC independence bungalow.
- All relevant aspects of Petran’s care package/care plan should be discussed.
- Petran should be made aware of how to contact his District nurse. His named nurse needs to contact his district nurse to discuss his specific needs.

Physical Healthcare

- Petran would like to see his scans with regard to his diagnosis.
- Petran would like information about the names, doses, reasons and side effects for all his medications.
- Petran needs to be informed of his new normal blood pressure.
- Petran’s sister helps with the management of his nails.
- Petran would like support in giving up smoking. He currently uses a nicotine patch but would like to stop using this.
- Pain score = 4 (shoulder) and 10 (neck), Petran reported experiencing pain in his shoulder and neck. He reported that pain does not interfere with his rehabilitation, and he would like more information on the type of pain he experiences and pain management. His pain medication (voltarol gel) requires review.
- Petran needs to be informed about how frequently he should take Nifedipine if appropriate and should be given his own supply to carry with him.
- Petran needs to be given information about what his normal vital capacity is and should be given an incentive spirometer for continuing use if required, shown techniques for secretion clearance and given a cough assist machine at discharge.

Daily Living Activities

- Petran needs to develop verbal independence skills in all aspects of daily living.
- Petran needs to start using a shower chair to help with his personal hygiene.
- Skin and Postural Management
- Petran needs to be made aware of what to do if he finds a red mark or pressure area and how to decide whether he should stay in bed or get up in his wheelchair to keep pressure off the area.
- Petran needs to commence asking staff to check his feet to avoid ingrowing toenails.
- Petran attended his first appointment at the Posture and Seating Clinic. He needs to commence instructing others to support him in carrying out regular pressure relief.

Bladder Management

- Petran uses suprapubic catheterisation.
- Petran should be given the opportunity to fully practice instructing others to change his catheter, leg bag and night drainage bag.
- The person who will maintain Petran’s catheter after discharge needs to be identified and given training.
- Petran needs to be fully informed of the size of the catheter he uses.
- Petran needs information on all issues relating to bladder-related problems.

Bowel Management

- Petran has reflex bowel function, which is currently performed on the bed at the NSIC.
- Petran needs information on all aspects of bowel management.
- Petran should be given information about where to seek help if he experiences bowel problems after discharge.
- Petran will require the use of a shower chair to manage his bowel care and bathing requirements.

Mobility

- LIKO hoist is used for transfers; a hoist will be needed for transferring on discharge.
- Petran needs to fully practice instructing others to transfer him.
- Petran is currently using a manual wheelchair and awaiting a powered wheelchair. Petran should be taught how to instruct about wheelchair skills for both of these.

Wheelchair and Equipment

- All issues relating to wheelchairs and cushions for discharge need to be addressed.
- Petran currently uses a standing frame. This will continue after discharge. The person who will assist with transfers after discharge should be identified and fully trained.
- Petran wears resting splints on his upper limbs, Procare ankle splints and an abdominal binder when standing.
- Petran highlights the following items that need to be ordered prior to discharge: bed, mattress, sliding sheets, hoist, slings, shower chair, cushion, positioning equipment, respiratory equipment and a standing frame.
- Petran needs to be fully informed where to obtain disposable leg bags, bed bags, catheters, disposables and disposable sheets.

Community Preparation

- Petran has not yet been out of the NSIC since his admission.
- Petran should be given the opportunity to practice accessing disabled toilets and public transport, instructing about laundry and using the OT garden.
- Petran needs to discuss returning to work with his OT.
- Petran needs to attend all the patient education sessions and be made aware of the patient information leaflets.
- It should be ensured that Petran’s family is invited to Friends and Family Day.
- Petran needs to be given information about how to apply for a “Blue” badge.

Psychological Health

- Petran is being provided with support from a clinical psychologist.
- All issues regarding intimacy and relationships must be addressed.
- Petran is aware of the support available from the family counsellor for his relatives.

## Appendix B. Goal Planning Practice in Spinal Cord Injury Rehabilitation, 2022

We are interested in understanding more about the use of goal planning in spinal cord injury rehabilitation. This is a survey of practice in the Centre/ Hospital in which you work. It consists of two parts: adults with spinal cord injuries and children and young people with spinal cord injuries. We may publish the results of this study as part of a peer-reviewed publication; if so, your responses will be anonymised. Linked with this, we are also interested in knowing who uses which versions of the Needs Assessment Checklist (Kennedy & Hamilton, 1999) and Child Needs Assessment Checklist (Webster & Kennedy, 2007). Please complete both sections if you serve both groups.

For a demonstration of the programme at Stoke Mandeville, please see https://youtu.be/yPt7cvqzSKk (accessed on 28 May 2022).

Name	Free text
Email	Free text
Centre at which you work	Free text
Country	Free text
* I work with adults with spinal cord injuries	Yes/No
We use the Adult Needs Assessment Checklist (NAC)	Yes/No
If you use the NAC, which version do you use?	Free text
We use a different holistic outcome tool that asks inpatients about their knowledge and skills as part of this tool	Yes/No
If you use another diagnostic tool, which one?	Free text
This other outcome tool can assess verbal rehabilitation skills, enabling inpatients with high levels of SCI to have maximum outcome gain	Yes/No
We ensure the information from the outcome/assessment tool is used to set rehabilitation goals	Yes/No
We repeat the assessment tool we use at or near the end of rehabilitation	Yes/No
We ensure the inpatient is always present at the multidisciplinary goal planning meeting	Yes/No
We include families within the multidisciplinary goal planning meeting, either through being present or being sent goal information after the meeting	Yes/No
We ensure all, or at least 4 members of the MDT, attend the goal planning meeting	Yes/No
We ensure multidisciplinary goal planning meetings take place regularly during rehabilitation, as needed but as a minimum an average of every 4 weeks	Yes/No
I would like to receive updates about the Stoke Mandeville Spinal Needs Assessment Checklist and Goal Planning Programme	Yes/No
I would be interested in knowing more about the Stoke Mandeville Spinal Needs Assessment Checklist and Goal Planning Programme, and/ or participating in research, please add me to your mailing list	Yes/No
* *The survey used to assess goal planning practice in the rehabilitation of CYPs with spinal cord injuries was identical in all respects apart from the fifth question, which read: ‘I work with children with spinal cord injuries’.*	

## Appendix C. NSIC Psychology Goals for Goal Planning Meetings

**NSIC Clinical Psychology Goals**

**GENERIC GOALS (Added to all patient SMS-NAC and GP notes)**

○ Patient to be supported by rehabilitation team to be able to manage the emotional demands of his/her injury
○ Patient to have regular contact with their keyworker and additional psychological support when needed.

➢ **Engagement in rehab**
  ○ Patient to be able to fully engage in rehab.
  ○ Psychological support to be provided to improve or maintain aspects of engagement.
  ○ Patient to continue to attend regular clinical psychology treatment/therapy sessions.
➢ **Distress and mood (Above threshold on SMS-NAC)**
  ○ Patient to have an introduction to the clinical psychology service.
  ○ Patient to attend therapeutic clinical psychology sessions as timetabled to focus on:
    ➢ Perceptions of disability;
    ➢ Anxiety;
    ➢ Coping;
    ➢ Etc.

**Other specific goal examples can be included:**

Self-advocacy

- Patient to be able to express their own needs and speak to their named nurse about these.
- Patients write their own goals before the next GPM.
- Treating clinical psychologist to attend a DST to provide support to the ‘Patient.’
- Adherence
- Patient to attend X amount of physio/hydro/OT etc sessions per week.
- To identify barriers to attendance for all weekly sessions.

Personal agency (Environment)

- Timetable to be written on patient’s whiteboard.
- Mobile phone to be positioned within reach.
- Patient to be involved with community discussions.
- Timetable to be amended to support choice and engagement.
- Attend My Health session on patient education and have a take-home leaflet.

Psychoeducation needs

- Patient to attend patient education sessions (managing adversity/pain management).
- To understand how anxiety might impact day-to-day activities.
- Patient to be informed about where to find information about managing anxiety.
- Identity
- Patient to be supported to think about how to reconnect with previous enjoyable activities.
- Upskill on assisted technology to re-engage with social networks.
- Patient to spend time identifying and exploring key values, priorities and aspects of life that are important and meaningful to him/her.
- Discuss pre-existing views of disability.
- Patient be supported to gain knowledge regarding sexual function, discuss impact of SCI/receiving care on their relationship.

Pain management

- Understanding triggers or factors relating to current pain.
- Discuss the management of pain.

Anxiety/Low mood

- Identify strategies to manage anxiety in the moment.
- Explore psychological techniques to manage anxiety.
- Think about weekend/evening activities and identify one activity to do each weekend day.
- Planning for timetable post-discharge.
- Social contact with family and friends.
- Discuss or share with a member of the team when you are having difficult thoughts/feeling low.
- Consider whether joint sessions with OT/PT would enhance rehab goals—where anxiety preventing participation/limiting functional range/fear of falls, etc.

Confidence

- Explore concerns about changed appearance.
- Begin to find out more about return-to-work schemes.

Cognition

- Patient to be offered cognitive assessment where appropriate and supported to understand the outcome.
- Patient to be supported to manage cognitive issues to promote engagement with rehab.

Sleep

- Explore psychoeducation.
- Discuss principles of sleep hygiene.
- Provide a Midlands SCIC booklet on sleep and bed rest if relevant.

Discharge preparation; Managing the transition

- Patient to be supported to prepare for the psychological/emotional demands of discharge.
- Patient to be referred to Back Up mentoring.
- Patient to attend My Health patient education session.
- To scope local community services and plan the first few weeks after discharge.
- Be given and have a discussion about ‘Making the Move’ leaflet.

PU/skin management

- Provide a Midlands SCIC booklet on sleep and bed rest if relevant.
- Learning needs, knowledge and skills to be considered as part of adherence.

Self-management

*Goals identified on the first page*

Engagement

*Goals identified on the first page*

Engagement with co-located services

- Patient to have information about liaison psychiatry clinic and to be referred as appropriate.
- Patient to have information about substance use services and to be referred as appropriate.
- Patient to have support to engage with psychiatry/substance use services.
